# An innovative approach to using both cellphones and the radio to identify young people’s sexual concerns in Kinshasa, Democratic Republic of Congo

**DOI:** 10.1186/2049-3258-72-21

**Published:** 2014-06-24

**Authors:** Gabriel Vodiena Nsakala, Yves Coppieters, Patrick Kalambayi Kayembe

**Affiliations:** 1 "Carnet de santé asbl", Communication Research and Health Promotion in DRC, Kinshasa, Democratic Republic of Congo; 2 Research Centre "Policies and Health Systems - International Health", School of Public Health, Université Libre de Bruxelles (ULB), Brussel, Belgium; 3 Department of Epidemiology and Bio Statistics, School of Public Health, University of Kinshasa, Kinshasa, Democratic Republic of Congo

**Keywords:** Emotional and sex life, Cell phone, Radio, Young people, Democratic Republic of Congo

## Abstract

**Background:**

As teenagers have easy access to both radio programs and cell phones, the current study used these tools so that young people could anonymously identify questions about sex and other related concerns in the urban environment of the Democratic Republic of Congo. The purpose of this healthcare intervention was to identify and address concerns raised by young people, which are related to sexual health, and which promote youth health.

**Methods:**

This healthcare intervention was conducted over a six month period and consisted of a survey carried out in Kinshasa. This focused on 14 to 24 old young people using phone calls on a radio program raising concerns related to sexuality. The radio program was jointly run by a journalist and a health professional who were required to reply immediately to questions from young people. All sexual health concerns were recorded and analyzed.

**Results:**

Forty programs were broadcast in six months and 1,250 messages and calls were recorded:

880 (70%) from girls and 370 (30%) from boys, which represents an average of 32 interventions (of which 10 calls and 22 messages) per broadcast. Most questions came from 15-19- and 20-24-year-old girls and boys. Focus of girls’ questions: menstrual cycle calculation and related concerns accounted for the majority (24%); sexual practices (16%), love relationships (15%) and virginity (14%). Boys’ concerns are masturbation (and its consequences) (22%), sexual practices (19%), love relationships (18%) and worries about penis size (10%). Infections (genital and STI) and topics regarding HIV represent 9% and 4% of the questions asked by girls against 7% and 10% by boys. Concerns were mainly related to knowledge, attitudes and competences to be developed.

**Conclusions:**

Concerns and sexual practices raised by teens about their sexual and emotional life have inspired the design of a practical guide for youth self-training and have steered the second phase of this interactive program towards supporting their responsible sexuality.

## Background

In the developing countries, about 60% of the population are under 25 years of age [1]. Health problems among adolescents (10–19 years) and young people (14–25 years) are basically socio-behavioral and require an answer adapted to their stage of development and to realities linked to their living environment [2,3].

In Africa, morbidity and mortality related to STI/HIV/AIDS and other problems of sexual and reproductive health of young people can be reduced by enhancing access to information, sex education, and reproductive health services.

The major role of healthcare providers in the dynamics of protecting young people against HIV/AIDS [4]; as well as of mass media (radio and television), which are acknowledged as the chief source of information – especially for sexuality issues – needs to be emphasized.

In the DRC, given their taboo nature, worrying issues of sexuality as well as intimate practices of teenagers and youth are rarely disclosed. This is because according to teenagers and youth these subjects should only be shared with trusted individuals who respect their privacy [5]. At the same time, they strongly need a listener-advisor for these concerns and an interpersonal approach to sex education by credible players [5]. So, a specific strategic approach guaranteeing discretion and even anonymity is essential to better tackle issues regarding support for and initiation to responsible sexuality. As a matter of fact, in the Congolese urban area, there are many opportunities to communicate with the youth, particularly via the community radio and television channels [6]. In parallel, access to and usage of cell phones have significantly grown in RDC [7], contrary to internet which is still expensive with limited accessibility. Given this easy accessibility and the impact of the media on the behavior of young people, we have asked ourselves if these two tools (cellphone and radio) might help significantly teenagers and youth in RDC urban areas, thereby improve their sex education. This research aims to identify and describe – through an innovative method combining the use of cellphone and radio – the questions, needs, expectations, and practices of teenagers in DRC urban areas concerning their sex and emotional life via youth healthcare programs.

## Methods

This is an exploratory, descriptive and analytical study using two communication tools referred to as the radio and the cellphone in the process of collecting data. It was carried out in a six month period, that is from June 12^th^ to December 10^th,^ 2011 in Kinshasa, DRC. The choice of the capital city results from the fact that it has a high number of audiovisual media (51 TV channels and over forty radio stations, all local) broadcasting from and for the city and its surroundings [6], as well as several phone operators in this environment [7].

### Information collection mechanism

Information is collected through an interactive radio show program in which 14-24- year-old adolescents and young people – along with other age range people participate by means of their cellphones.

This information collection mechanism was inspired by the noticeable sound participation of young people, via SMS, in radio or TV entertainment programs to dedicate music to third parties – regularly broadcast in the DRC.

The program “S’il vous plait docteur” or *Please Doctor* lasts 90 minutes and is broadcast three times a week from 6.00 to 7.30 (PM) on B-One, which is among the radio stations with the highest youth and teenagers audience (according to an opinion poll carried out in Kinshasa [8]). This radio station broadcasts on 87.8 FM and is easily picked up throughout Kinshasa and its surroundings. There are two ways to take part in the program: i) the participant can send a text message (SMS) before, during, or after the program and/or ii) live calls during the program. Participants must first state their age, their place of residence and, optionally, their name or nickname. This allows for their anonymity to be preserved, while processing answers in accordance with that above profile. The first half of the program is devoted to addressing issues related to the growth and development of girls and boys, and the second part is reserved for answers to concerns that are raised by teens. Sometimes, the program only dealt with the questions and worries teens raised and from the third month of intervention, topics were dealt with thematically in line with the major concerns expressed, such as menstrual and menses trouble, STIs, HIV/AIDS, hygiene, etc.

### Key actors and targets of the program

The program was jointly produced by a journalist and a doctor with the help of a technician. The (lady) journalist steered the broadcast, centralized the SMS messages sent, and aired the concerns of the listeners as they had given them. The physician, who is a qualified public health doctor with an additional degree in communication and training on youth matters, answered personally each listener relating to the different concerns addressed by SMS and live calls concerns. The technician ensured the broadcasting and recording of all productions for repeated broadcasts and archiving.

The program was purposefully conducted in French for schooled teenagers. Participation in the program was made possible for everyone. However, the data taken into account and analyzed are exclusively those drawn from unmarried young boys and girls whose the age ranges from 13 to 28 years, divided into 4 age groups: 14 years or under, 15–19 years, 20–24 years, and 25 years or over, who all live in Kinshasa. The program targeted primarily adolescents (10–19 years) and young people (14–25 years) (categories defined by WHO) who need support before and during puberty in order not to be surprised by troubles linked to inexperience in sexuality such as early pregnancies and STI/HIV/AIDS, and even beyond puberty – in order to give a sense of responsibility for a healthy future life [2,3,9-11].

### Data analysis

All the SMS messages received during the program implementation were chronologically recorded in a notebook detailing all socio-demographic variables of participants.

From the synthesis of all the records, it was possible to proceed not only to the descriptive statistical analysis of data, but also to a qualitative analysis of the contents and discourse of the participants by the research team. The descriptive and interpretative method of examining the contents of the listeners’ messages and discourse allowed pinpointing essential issues emerging from raw data which were classified in accordance with gender and age groups. Organized thematically, analysis of discourse and the construction of expressions connected with the object of study allowed classifying SMS messages as questions, concerns, practices, or need for knowledge.

### Ethical considerations

The survey is part of a series of studies that aims to improve communication strategies for the prevention of HIV/AIDS and unwanted teenage pregnancies. Its protocol was approved by the Ethics Committee of the Public Health School of Kinshasa University. However, participation in the survey was voluntary with a maximum guarantee of anonymity, which was being clearly indicated during each broadcast, right after the objective had been set up.

## Results

In all, 40 programs were broadcast during the study. During the first three months, the show was especially focused on adolescence, puberty, growth and development of girls and boys. According to the nature of the questions posed, the subjects gradually diversified to the menstrual cycle and its troubles, unwanted pregnancies and terminations, STIs, HIV/AIDS and personal body hygiene. In total there were 1,250 messages and calls, of which 880 (70%) from girls and 370 (30%) from boys. The average is 32 messages per broadcast, that is 10 calls and 22 messages. All participants came from Kinshasa 24 townships with a predominance of about 65% of participants from mainly residential townships (Ngaliema, Limete, Gombe, and Mont-Ngafula).

Table 1 shows the breakdown of listeners whose concerns were recorded according to sex and age group.

**Table 1:** Profile of young Congolese who took part in radio programs in Kinshasa, according to sex and age group (June – December 2011)

**Sex**	**Age groups**				**Total n (%)**
	**14 years and less**	**15-19 years**	**20-24 years**	**25 years or over**	
	**n (%)**	**n (%)**	**n (%)**	**n (%)**	
**Girls**	38 (3%)	399 (32%)	318 (25%)	125 (10%)	880 (70%)
**Boys**	8 (0.7%)	116 (9.5%)	165 (13.3%)	81 (6.5%)	370 (30%)
**Total**	46 (4%)	515(41%)	483(38%)	206 (17%)	1,250 (100%)

Girls in the 14–19 and 20–24 age groups were more numerous in expressing their concerns, whilst for boys, the most participative age group was the 20-24-year olds.

### Major worries of young people

The different worries of young people are divided into 3 points: i) concerns that are common to girls and boys are: emotional relationships, intimate practices (15.5% for girls and 19% for boys), genital infections and HIV issues (9.4% for girls and 7% for boys), condom use (4.4% for girls and 10% for boys), pregnancy and fecundity (2.6% for girls and 1.6% for boys); ii) concerns specific to girls revolve around issues related to menses and their problems (24%), virginity (14%), abortion (4.5%), breast size (3%), personal hygiene (3%); iii) boys’ particular worries are specifically connected to masturbation (22%) and penis size (10%). Table 2 lists different themes of boy’s and girls’ worries according to age groups.

**Table 2:** Different themes of boys’ and girls’ worries according to age groups (June – December 2011)

**Major concerns**	**Distribution by sex and age groups**								**Total**	
	**14 years or less (N)**		**15-19 years (N)**		**20-24 years (N)**		**25 years and over (N)**			
	**Girls N(%)**	**Boys N(%)**	**Girl N(%)**	**Boys N(%)**	**Girls N(%)**	**Boys N(%)**	**Girls N(%)**	**Boys N(%)**	**Girls N(%)**	**Boys N(%)**
**Menstrual cycle**										
Troubles and menses calculation	13(1,5)	0(0,0)	105(12,0)	2(0,5)	71(8,0)	6(1,5)	20(2,5)	8(2,0)	**209(24**%**)**	**16( 4**%**)**
**Emotional life**										
Love Relationships	17(2,0)	0(0,0)	79(9,0)	27(7,5)	29(3,1)	28(7,8)	8(0,9)	10(2,7)	**133(15**%**)**	**65(18**%**)**
Virginity	0(0,0)	0(0,0)	57(6,5)	0(0,0)	42(5,0)	0(0,0)	22(2,5)	0(0,0)	**121(14**%**)**	**0(0**%**)**
**Practices related to sexuality**										
Intimate (sex) Practices	4(0,5)	0(0,0)	63(7,3)	16(4,5)	49(5,5)	35(9,5)	19(2,2)	18(5,0)	**135(15,5**%**)**	**69(19**%**)**
Intimate Care	1(0,1)	0(0,0)	13(1,5)	0(0,0)	11(1,1)	0(0,0)	3(0,3)	0(0,0)	**28(3**%**)**	**0(0**%**)**
Masturbations	0(0,0)	4(1,1)	6(0,7)	28(7,7)	7(0,8)	34(9,4)	1(0,1)	14(3,8)	**14(1,6**%**)**	**80(22**%**)**
**Genital infections and HIV matters**										
Infections	0(0,0)	0(0,0)	36(4,1)	8(2,0)	29(3,3)	12(3,2)	17(2,0)	07(1,8)	**82 (9,4**%**)**	**27(7**%**)**
Condomns/ HIV(use)	0(0,0)	2(0,5)	14(1,5)	12(3,0)	18(2,0)	19(5,0)	8(0,9)	6(1,5)	**40 (4,4**%**)**	**39(10**%**)**
**About conception**										
Abortions	0(0,0)	0(0,0)	5(0,6)	0(0,0)	20(2,2)	0(0,0)	15(1,7)	0(0,0)	**40(4,5**%**)**	**0(0**%**)**
Pregnancies/ Fertility	0(0,0)	0(0,0)	7(0,6)	0(0,0)	14(1,5)	6(1,6)	4(0,4)	0(0,0)	**25(2,6**%**)**	**6(1,6**%**)**
**Body shape (about)**										
About breasts (size)	0(0,0)	0(0,0)	11(1,2)	0(0,0)	14(1,6)	0(0,0)	2(0,2)	0(0,0)	**27(3**%**)**	**0(0**%**)**
Penis size	0(0,0)	2(0,5)	0(0,0)	15(4,0)	0(0,0)	11(3,0)	0(0,0)	8(2,5)	**0(0**%**)**	**36(10**%**)**
**Others issues**^**a**^	3(0,3)	0(0,0)	4(0,5)	3(0,5)	13(1,5)	6(1,5)	6(0,7)	4(1,0)	**26(3**%**)**	**13(3**%**)**
**Total**	**38(5**%**)**	**08(2**%**)**	**399(45**%**)**	**116(31**%**)**	**318(36**%**)**	**165(45**%**)**	**125(14**%**)**	**81(22**%**)**	**880(100**%**)**	**370(100**%**)**

^a^Others issues = Inbreeding relationships, Homosexuality, Pornography ,Cysts / *Myoma.*

In analyzing the subject of the sent messages, concerns were categorized according to their contents. Thus, period matters were categorized according to knowledge about calculation of menstrual cycle, to attitudes to be adopted in case of period troubles and to what to do (skills) in the event of pain. Worries related to emotional relationships showed a need for knowledge (illustrations: the possible hazards of falling in love at 14 years of age; or of knowing what makes you feel like loving a boy, or of the age difference between a boy and a girl in an emotional relationship). Attitude issues were also raised on this theme. For instance, how to behave if you want to have an emotional relationship devoid of sex or how to deal with dating request from the other sex? Sexual practice issues were mainly centered on the consequences of some sexual practices such as sodomy, fellatio, and vaginal touching the study population indulges in. Other practices such as lesbianism worried girls as well. The issue of virginity was also raised, not only in terms of the couple sexual life benefits, but also its impact on certain sexual practices (touching, previous rape) or in case of prolonged celibacy.

Among boys, knowledge questions are varied and often directed towards such topics as consequences of frequent sexual intercourses, HIV contamination through saliva, calculation of girls’ cycles, the beginning of the fertilizing power of sperm. This is supplemented to questions pertaining to attitudes to be adopted in order to have a healthy emotional life and how to resist girls’ charms. Masturbation is central to questions by boys of different ages. Given that many boys indulge in it, knowing its consequences is also vital. Moreover, boys are preoccupied by the way to fight it.

Whether girls or boys, one concern regards self-esteem concerning anatomy and hereditary constitution. Some girls are really worried by the size of their breasts which they consider being either too small or too big or deformed. Many boys fear the reduced size of their penis that they hope will get bigger if possible.

Adult participation was not overlooked. They brought up knowledge issues (i.e. contraception measures), but mostly attitudes one should adopt in the light of their offspring’s development during puberty, as well as the need to improve their expertise for good sex education.

## Discussion

This survey seeks to describe, in the context of DRC urban area, the intimate worries, questions, needs, expectations, and sexual practices of teenagers and young people. This is an innovative approach which has allowed the collection of very sensitive data related to the privacy of participants to which there is little access through the common channels [5].

In spite of its being essentially exploratory, this study has its limits. It only involved one category of young people: youngsters from urban area speaking French and in possession of a cellphone and radio with ability to send an SMS. Although some young people have mobile phones with a built-in radio, others however, do not, given the economic context of the DRC. Even if it is possible to use a family radio, sudden blackouts and the difficulty to change batteries can prevent some young people from benefiting from this program. However, frequent reruns of recorded broadcasts and the possibility of using one radio by several listeners and sharing the same cellphone among friends can help to reduce shortcomings.

### On the interest of the approach used in the survey

This study has not only enabled the collection of sensitive data concerning the sexual and emotional lives of young people. It also is a health promotion intervention. It constitutes a highly original means that can effectively contribute to young people’s sex education, by exploiting the contribution of two communication tools that are underused for this purpose.

By using a participative method in the promotion of public health seeking the involvement and participation of adolescents and young people to better monitor their health [12], the program effectively put the benefit in the center of the action. As such, the answers to the needs expressed were personalized according to type of concern expressed by the listener. In practice, we could fall back on several complementary approaches according to the case [13]: the injunctive or persuasive approach that seeks the systematic modification of an individual’s conduct, and the informative and empowering approach which stimulates people to become aware of what is good for them.

Used to maintain social links, new information and communication technologies (NICTs) are at the heart of mechanisms upholding human development. They can enable reducing populations’ inequalities in the access to health education and so contribute to the improvement of living conditions. Outcomes on individuals’ welfare were demonstrated, regardless of the individual’s living conditions [14,15]. The cellphone is present in households and in different services. It is currently an indispensable tool for all segments of the population (both literate and illiterate) – the young and adolescents, both in urban and rural localities [15]. As a mass communication medium, the radio offers some flexibility, great selectivity (targeted according to age range), and a widely reachable audience [16]. Given the built-in frequencies – among other functions some young people look for – in cellphones, such medium is very popular and allows youngsters to listen, discretely, and to take an active part in a program dealing with sensitive topics without being embarrassed.

The use of the cellphone to take part in radio programs guarantees confidentiality and the resulting interactivity helps to influence behavioral attitudes and choices. The fact of personalizing information according to the problems expressed also allows a quick and direct action for the person through the advice and guidance of a service trained for such matters. In many mid human development and low income countries, the contribution of cellphones in the improvement of health standard is real [1]. Their widespread availability has offered opportunities to boost access to information connected to health, to educate adolescents and young women, and to improve the quality of healthcare offered by health departments [1].

The high number of messages in comparison to calls is due (i) to economic reasons probably, and (ii) to the freedom of asking personal questions which, if expressed in calls, would be embarrassing to oneself and to others. Messages allow displaying one’s emotions and intimacy. Thus, one can, at any moment and without social constraint, say anything, say things that one would not utter orally – and even deep secrets [17]. Likewise, SMS messages permit contacting the person directly, without the risk of interception by a third party [17]. Furthermore, the opportunity to have open-hearted exchanges on the radio on issues of concern with an available specialist encourages their use. In short, the mechanism exploited strengthens the unity of actors from diverse sectors (health, media, sound engineer, telephone operator) in a perspective of reorientation of services as advocated by the Ottawa Charter for Health Promotion [18]. In so doing, we could expect to influence other media to fill the identified shortcomings in sexual education of the young people and adolescents. This would affect their attitudes and viewpoints and enable some social norms to evolve. Social norms are the behavior that other members of the social group expect from a person; so, they are community attributes. A media strategy can intervene in two ways: either by changing the current community norm or by trying to modify the perception of the norm [19]. Given that the radio can reach a wide public instantly and that the cellphone is accessible to many young people, this mechanism also contributes to reducing social disparities in terms of access to good information. As such, the approach used prevents the false or wrong ideas that the youth has and corrects them if necessary. It also enables ascertaining the fields in which teenagers and young people wish to deepen their knowledge. Lastly, it facilitates the identification of risky practices and attitudes towards sexual and reproductive health issues.

#### On young people’s worries, questions, expectations, and sexual practices

The different youth preoccupations were identified. They differ according to sex and age groups. Young people and adolescents’ questions and worries relate to knowledge, attitude, practice and self-esteem as regard sexual and reproductive health.

The particularly high participation of girls in the 15–19 and 20–24 age groups is justified by their more advanced maturity than boys’ [2]. The higher participation in this study by young people from residential townships is probably justified by their relatively higher social living standard that might have facilitated the availability of call credit and the culture of listening to the radio in these areas. The focus on the menstrual cycle and masturbation intricates the youth in many developing countries such as Sri Lanka where similar preoccupations were expressed by both girls and boys [20]. In the DRC, young people are particularly concerned with different issues they are habitually deprived of during outreach activities in the community milieu. In fact, most of the issues that worry them are rarely dealt with in the mass media thereby leaving for debate in family talks. Besides, although some of these issues are contained in school textbooks on youth mobilization against HIV/AIDS [21], training paired educators in reproductive health [22]; as well as on high school sex education [23], these themes are often hardly taught interactively and thoroughly.

Although the above are not reliable contraceptive methods, a good understanding of the menstrual cycle by young people (girls and boys) can influence their decision making attitude towards accepting or refusing sexual intercourses. And when these are coupled with an appropriate use of protective/condoms, unwanted pregnancies’ prevention would be better reinforced, and can contribute to reducing high maternal mortality in the DRC [24]. Emotional issues are of particular interest for girls and boys during their youth. Rather than being a step to discovering a partner and showing friendship, relationships between girls and boys in Kinshasa are oriented towards extramarital sexuality [25]. The analysis of relationships between girls and boys in Kinshasa reveals that the average time between the first contact and the first sexual relationship between two young people is two weeks [26]. And once it has begun, sexual activity is intense and often involves more than one partner [25], based on seeking pleasure and material benefits [5]. In this context, an effort should be made to fight the effects of rumors concerning girls’ sexuality, which profoundly upset them in this regard [27]. Similarly, the issue of masturbation – a great concern among boys – should be addressed radically to help them overcome this concern. Worries about breast and penis size respectively expressed by girls and boys deserve special attention in the education of young people since they can significantly impact their self-appreciation and self-esteem needed to build their personality [22]. In addition, the low proportion of questions about HIV/AIDS and STIs recorded among both boys and girls can be explained by the fact that the latter are not yet aware of the risk and vulnerability involved and so do not feel directly affected by these issues. Yet, in a country where HIV prevalence among young people is higher than the national average (3.8% compared to 2.5%) [28] with 28.4% of teenage pregnancies and nearly 20% maternal mortality [29], young people in the DRC ought – more than others – to be provided with the necessary skills for better prevention of HIV and unwanted pregnancies.

The identification of these concerns is important in terms of public health because it consistently helps support young people who should be provided with the skills that allow them to decide responsibly [2,3,11,30]. This approach also helps to support parents, to whom the program offers an opportunity to impact sexual education within families, while urging them to lift the taboo on sexuality issues with their children. At the same time, it allows the self-training of young people’s *coaches* who are responsible for taking care of them in their communities (community associations, religious associations, teachers, and so on).

## Conclusion

In perspective, the results of this survey are indicative of the need to adapt interventions in accordance with young people’s age, gender and stage of development. At the same time, they call for the promotion of sex education interventions which should fearlessly and freely tackle sexuality and work different strategies, taking into account the fact that boys’ and girls’ needs are diverse in a specific socio-cultural context [30] that should contribute to personal and social behavior change of young people.

In the light of the data reported in this study, a practical self-training guide for teens clearly addressing the issues raised about their sexual and emotional life was designed, and the second phase of this interactive program is oriented towards encouraging responsible sexuality among the youth in DRC.

## Competing interests

The authors declare that they have no competing interest to this work.

## Authors’ contributions

GVN conceived the survey, analyzed data, and drafted manuscript. YC and PKK oriented data analysis and reviewed the manuscript. All the others approved the final manuscript.

## References

[B1] UNFPA Sexual and Reproductive Health for All. Reducing poverty, advancing development and protecting human rights 2000

[B2] World Health Organization Adolescent Health Programme. Guidance on health providers 2005 Geneva: Department of Children Health and Adolescents Development

[B3] World Health Organization Health and Adolescent Development for effective programming. Report of a study group programming for adolescent health WHO, UNFPA, UNICEF 1999 Geneva: WHO

[B4] WHO Protect young people against HIV and AIDS, The role of health services 2006 Geneva: WHO

[B5] Nsakala VG Coppieters Y Kayembe KP Lapika DB Gomis D Adolescents’ and youth people’s perceptions of sexual and reproductive health in Democratic Republic of Congo Revue Santé publique 2012 24 5 403 415 23472982

[B6] Frère M-S The Congolese Media environment: Inventory and challenges 2008 France and British International Cooperation 167

[B7] United Nations Programme for Development Undertake the benefit of all. Study cases 2006 Democratic Republic of Congo: Celtel and Celpla; UNDP

[B8] Experts Report of audiovisual hearings media in Kinshasa DRC. 1^st^ term 2011

[B9] World Health Organization Adolescent Health, Strategy of the African region 2001 WHO-Afro

[B10] Measuring communication Youth in Sub-Saharan Africa data and charts on sexuality and reproductive health 2001 Population Reference Bureau 44 Report

[B11] WHO, UNFPA, UNICEF Action for adolescent health. Recommendation of a joint study group 1997 Geneva: WHO

[B12] World Health Organization Ottawa Charter for Health Promotion, Toward a New Public Health 1986 Ottawa, Canada: First International Conference on Health Promotion

[B13] Bury J Health education: concepts, issues, planning 1988 Deboeck University

[B14] UNDP - INS Niger Impact of mobile cellphone on the lives of users and market participants 2009 Investigation Report

[B15] Modandi M Development of mobile telephony and social links in Africa, the case of Gabon. PhD thesis 2005 Communication Institute, Université Lumière Lyon 2 301

[B16] Bontemps R Cherbonnier A Mouchet P Trefois P Communication and health promotion. Theoretical, methodological and practical aspects 2004 2 Question santé asbl

[B17] Martin C Cellphone among youth and their parents What legitimation for uses? Second Workshop of Marsoui 2003 Brest: ENST Bretagne

[B18] International Union for Education and Health Promotion Shaping the future of health promotion 2007 Vancouver (Canada): CCHPR 10

[B19] Renaud L Rico de Sotelo C Kane O International Communication and health homology approaches Communication Horizons practices and research, under the direction of Pierre Mongeau and Johanne Saint -Charles 2005 Montreal: PUQ 30 51

[B20] Agampodi SB Agampodi TC Piyaseeli UKD Adolescents perception of reproductive health care services in Sri Lanka BMC Health Serv Res 2008 8 98 1 8 18454869 10.1186/1472-6963-8-98 PMC2386785

[B21] DRC Ministry of Health, National Program for the Fight against AIDS- PNLS Guide to social mobilization for youth for HIV/AIDS prevention 2004

[B22] Diallo I Tshiofukila B Mampaka A Training on Adolescent Prevention STI-HIV/AIDS and other issues of reproductive health through education in life skills. The peer educator Guide 2007 121

[B23] The Central Service for life Education, national NGO-DRC Syllabus of life education, primary and secondary school levels (all options) 2008

[B24] Nsakala VG Coppieters Y Vulnerability and risk profiles related to reproductive health of youth and adolescents during the last decade in DR Congo. Revue Congo- Afrique51st year. No. 466, p. 427 (39), June- July-August 2012

[B25] Kalambayi Banza B Youth Sexuality and risky sexual behavior in Kinshasa (DRC) 2007 Department of Population Science and Development. Institute of Demography, Catholic University of Louvain. Academia Bruylant 378

[B26] Fund United Nations Children’s Fund UNICEF, School of Public Health Kinshasa Participatory analysis of the health, development and participation of adolescents with adolescents, the method of narrative research 2007 Kinshasa: SPHK 45 Research Report

[B27] Ministry of Health, National Program for Adolescent Health Inventory of rumors about the reproductive health of young adolescents 2008

[B28] Multisectoral National Program for the Fight against AIDS PNMLS Epidemiological Survey Report of HIV/ AIDS 2011

[B29] The National Institute of Statistics and the United Nations Fund for Children Multiple Indicator Cluster Survey in Democratic Republic of Congo (DRC MICS- 2010) 2011 Final Report

[B30] UNESCO, UNAIDS Manual for information, education, culturally appropriate communication Design and dissemination. Division of Cultural Policies and Intercultural Dialogue 2002 UNESCO

